# Antibacterial Effects of Persica Mouth Wash on *Helicobacter pylori* Growth

**DOI:** 10.4103/0974-777X.62864

**Published:** 2010

**Authors:** Omid Teymournejad, Morteza Sattari, Ashraf Mohebati Mobarez, Davood Esmaeili

**Affiliations:** *Dept of Bacteriology, Center of molecular Biology research, Tehran, Iran*; 1 *Tarbiat Modares University, Faqulty of Medical sciences, Tehran, Iran*; 2 *Baghyatallah University of Medical sciences, Center of molecular Biology research, Tehran, Iran*

Sir,

The resistance and toxicity of the current antibacterial agents and cost of the treatment has led to the development of more harmless and cheap treatment methods.[1,2] Since ancient times, medicinal plants have been used for the treatment of bacterial infections.[1] More studies are performed on the effects of different plant agents on different microorganisms and new antibiotics have entered the market based on the plant ingredients. Oral hygiene measures have been practiced by different populations and cultures around the world since antiquity. Chewing sticks were used by the Babylonians some 7,000 years ago.[3] It was later used throughout the Greek and Roman empires and has been used by Jews, Egyptians and Muslims. Today, they are used in Africa, Asia, the Eastern Mediterranean region and South America.[1,2] In the present study, we have investigated the effect of a plant mouth wash called Persica on the growth of *Helicobacter pylori*.

*H. pylori* was isolated from 10 clinical and environmental samples. Isolates from water and biopsies were confirmed by culturing, rapid urease and morphological test by Gram staining. We used *H. pylori* 26,695 as standard.

Persica mouth wash is prepared from *Salvadora persica* extract. It is composed of three medicinal plants: Miswake, a member of the Salvadoraceae family, Mint and Yarrow, and is available together with special Tannin (Poursina Laboratory, patent. Tehran, IRAN).

We poured different dilutions (1/2, 1/4, 1/8, 1/16, 1/32, 1/64) of the mouth wash in Brucella agar supplemented with 7% sheep blood, fetal calf serum, polymixin B, amphotericin B, terimetoprim and vancomycin. Then, we cultured the supplemented Brucella agar containing different dilutions of mouth wash with clinical, environmental and standard isolates of *H. pylori*.

Antibacterial activity of Persica mouth wash against *H. pylori* growth emerged on 1/16 dilution of stock and completely prohibited *H. pylori* growth. No difference was seen between the inhibitory effect of clinical, environmental and standard *H. pylori* [Table 1].

**Table 1:** Inhibitory effects of Persica on *Helicobacter pylori* strains

Samples	1/2	1/4	1/8	1/16	1/32	1/64
Standard sample	+	+	+	-	-	-
Biopsy samples	+	+	+	-	-	-
Environmental samples	+	+	+	-	-	-

+ , growth of H. *pylori*;

- , inhibition of growth

*H. pylori* was separated from dental plaque and saliva by the polymerase chain reaction (PCR) technique. Also, a high activity of urease in saliva with a positive PCR test was shown at the same time.[4] Mouthwashes are very useful in reducing microbial plaques.[5] One of the *H. pylori* infection sources is mouth and dental plaque.[4] Eradication of this bacterium from the mouth and dental plaque can prohibit bacterium reinfection in the stomach and remove one of the main bacterium sources.

Adenotonsillar hypertrophy is known to be related to the presence of the bacterium in the mouth because of tissue stimulation by urease and cag A bacterium agents.[6]

Because of the presence of the bacterium in dental plaque and weak penetration of antibiotics on dental plaque, the bacterium colonize in the stomach again and infection reappears after a while following taking antibiotics and removing the stomach infection.[7]

Reappearance of *H. pylori* is one of the concerns for which many theories have been mentioned to explain the mechanism.[7]

Using mouth wash is one of the ways to remove *H. pylori* in dental plaque. Mouth wash must have special specifications so that it would be least effective on the mouth and teeth flora and reduce the amount of harmful bacteria in that area.[5]

In the present study, we found that Persica mouth wash strongly inhibits *H. pylori* growth in the mouth.[1]
